# Foreign-language skills of student paramedics studying undergraduate Paramedic Science in the UK: a nationwide cross-sectional exploratory study

**DOI:** 10.29045/14784726.2025.3.9.4.37

**Published:** 2025-03-01

**Authors:** Owen Finney

**Affiliations:** North East Ambulance Service NHS Foundation Trust; University of Sunderland ORCID iD: https://orcid.org/0000-0003-3744-2710

## Abstract

**Introduction:**

Communication is essential in healthcare, but language barriers between patients and clinicians can hinder care quality, especially as the UK sees an increasing number of non-native English speakers. The 2021 UK census revealed that 5.1 million people do not speak English as a first language, with many having limited or no proficiency. Despite these trends, little research has been carried out to explore the experiences of these individuals in pre-hospital care, where language challenges often arise. UK paramedic education includes communication training, but foreign-language learning is not part of most curricula. Some universities offer optional language modules, yet there is no data on how many paramedics or student paramedics speak additional languages. Clinicians with foreign-language skills could enhance patient satisfaction and reduce communication barriers, but there is a lack of pre-hospital evidence in this area. This exploratory study aimed to capture the foreign-language skills of student paramedics in the UK, addressing a gap in the literature and laying the groundwork for future research.

**Methods:**

This exploratory study employed a quantitative, cross-sectional design using an online survey distributed to student paramedics across 24 higher education institutions in the UK during April and May 2024. The survey captured demographic data and language competencies, with descriptive statistics used for analysis.

**Results:**

Out of 105 respondents, 73 were female (69.5%), and the mean age was 23.65 (± 8.25). Over half (53.3%) reported proficiency in at least one foreign language, predominantly languages spoken in Europe, such as French (48.2%), Spanish (35.7%) and German (21.4%). Most respondents had beginner-level skills (64.3%), with no formal language training in their programmes. However, 57.7% expressed interest in studying an optional foreign-language module.

**Conclusion:**

This study found that over half of student paramedics possess foreign-language proficiency. The majority of the sample indicated a desire to study a foreign language if given the opportunity during their paramedic training. Given the increasing linguistic diversity in the UK, integrating language modules into paramedic education could enhance patient care and satisfaction. Further research is needed to explore the feasibility of such training and its impact on pre-hospital care outcomes for non-native English speakers.

## Introduction

Communication makes up a vital part of any healthcare interaction between patients and clinicians (Willis & Peate, 2024). It is well understood that language barriers have a negative impact on the quality of healthcare, and these barriers commonly occur between healthcare providers and patients when the two groups do not share a native language (Al Shamsi et al., 2020). The latest UK census estimated the UK population at mid-year 2021 to be 67.0 million (Office for National Statistics (ONS), 2022a). Out of this population, there is a wide array of first languages spoken, with most foreign languages increasing in the 10-year period from 2011 to 2021 (ONS, 2022b). For example, the number of Spanish speakers rose from 120,000 to 215,000 between 2011 and 2021 in the UK. Of the speakers that did not speak English as a first language (5.1 million people), 17.1% (880,000) could not speak English well, and 3.1% (161,000) could not speak English at all (ONS, 2022b).

Undergraduate paramedic courses in the UK typically occur over three years, with modules that incorporate life sciences, practical skills and other proficiencies, such as interpersonal communication and leadership (College of Paramedics (CoP), 2019). Communication skills are taught and developed through methods such as real human interactions during placements and simulation (Mangan et al., 2022). Learning foreign languages is not part of standard university paramedic programmes and is not mentioned in the CoP educational guidance; however, some universities offer extra-curricular foreign-language modules at a university-wide level (CoP, 2019; Newcastle University, 2023).

There are clinical benefits for clinicians that can speak foreign languages, such as improved patient satisfaction (Ngo-Metzger et al., 2007). Furthermore, language learners receive personal benefits, such as improved cognition (Bialystok, 2017; Kroll & Dussias, 2017). A UK governmental report found that UK citizens lack foreign-language skills in relation to meeting the demands of the adult workforce (Shepperd, 2021).

A narrative review in 2015 found that there was a lack of evidence exploring language barriers in pre-hospital care (Tate, 2015). Furthermore, there is no specific literature exploring how many languages are spoken among paramedics, ambulance staff or student paramedics in the current evidence base. Widening the literature search, one study found that 42% of physicians practising in California reported having fluency in at least one language other than English (Moreno et al., 2010). As foreign-language competency has not been explored in the pre-hospital setting in the UK, and there is a clear increase in the UK population with limited English proficiency, this exploratory study aimed to capture the foreign-language skills of the student paramedic population in the UK as a start in addressing this gap in the literature.

## Methods

The aim was to conduct an exploratory study to capture the foreign-language skills of the student paramedic population in the UK.

The objective was to quantify the number of foreign languages spoken among the student paramedic population in the UK and to describe which specific languages are spoken.

### Research design

The study was an exploratory study using a quantitative, cross-sectional design, employing an online survey to generate quantitative data to achieve the research aim and specific research objectives. Table 1 contains a list of the definitions used for this study.

**Table 1.** Key terms and definitions.

Term	Definition
Foreign language	A foreign language is a language that is not an official language of, nor typically spoken in, a specific country (Moeller & Catalano, 2015).
Official languages in the UK	The official language of the UK is a complex subject, as there is ambiguity around the topic (Mac Síthigh, 2018). In our study, we explained to the participants that the UK has several official and recognised languages: English (de facto across the UK), Welsh (official in Wales), Scottish Gaelic (official in Scotland) and Irish (recognised in Northern Ireland), as well as other recognised languages, such as Ulster Scots and Scots (Mac Síthigh, 2018).
CEFR	The Common European Framework of Reference for Languages (CEFR) was used to define language competence, which is an international standard for describing language ability (Cambridge English, 2024).

### Study setting

The study aimed to capture participants from the entire UK student paramedic population in higher education institutes of England, Wales, Scotland and Northern Ireland.

### Sample size and participant recruitment

A convenience sampling approach was implemented by contacting each Health and Care Professions Council (HCPC)-approved paramedic science course directly by email. Course leaders were sent an email by the principal researcher with details of the research; the email asked course leaders to disseminate a link to the course to the respective students. Out of the 29 paramedic courses identified, successful contact was made with 24 course leaders, who agreed to disseminate the research with their students.

### Inclusion criteria

The study included student paramedics studying an HCPC-approved paramedic science programme (BSc) at any higher education institute in the UK. The students could be at any stage within their paramedic programme of study.

### Exclusion criteria

Exclusions were made for paramedics, or other qualified ambulance staff, who were not student paramedics studying at a higher education institute. Those undertaking paramedic apprentice programmes were excluded, as this study solely focused on student paramedics studying undergraduate university programmes at higher education institutions, that is BSc degrees. MSc paramedic science students were also excluded.

The study focused on this specific paramedic student demographic, as there was limited information available at the time of the study to find all the relevant apprenticeship and Master’s-level courses that exist in the UK.

### Addressing bias

The research hypothesised that this design may be prone to self-selection bias due to the nature of the research. To mitigate against this, within the recruitment email it was emphasised that the survey was anonymous and would not take long to complete.

### Data collection

The data was collected between April and May 2024 using an online survey made on Qualtrix. The survey comprised two sections, with a total of 11 closed questions, averaging a 3-minute completion time, which explored the demographic details and language competence of the student paramedics. Table 2 contains a full breakdown of the quantitative variables that were collected.

**Table 2.** Data variables and their measurement collected by survey.

Variable	Measurement
Age	Age in years
Sex	Male, female, non-binary, other
Region	List to select from defined regions of the UK
Languages spoken at a basic level as per the CEFR scale	List*, with ‘other’ free-text selection if not listed
Languages spoken at an intermediate level as per the CEFR scale	List*, with ‘other’ free-text selection if not listed
Languages spoken at an advanced level as per the CEFR scale	List*, with ‘other’ free-text selection if not listed
Previous study of a foreign language during undergraduate paramedic university training	Yes or no answer
Opinion of studying an optional 10-credit foreign-language module while training to be a paramedic	Yes, no or maybe answer

*Language list used can be found in Supplementary 1.

### Data analysis

The data was exported from Qualtrix directly to Microsoft Excel and was summarised and analysed using descriptive statistics within the application. No inferential statistics were performed.

### Ethical approval

Ethics approval was gained through the University of Sunderland’s ethics process (reference number: 024975). This study did not require Health Research Authority approval, due to the nature of the study. The study was conducted as per the ethical approval, with no issues reported during the study. The data was managed as per the ethical approval.

The definitions provided in Table 1 were all included in the survey for participants.

## Results

In total, 105 student paramedics responded to the survey. A breakdown of the results can be seen in Table 3. The majority of respondents were female (69.5%), the mean age of respondents was 23.65 (± 8.25) and respondents were mainly from the regions of the North East of England, Scotland and Yorkshire.

**Table 3.** Overall results (n = 105).

Variable	n (%)
**Sex**	
Male (%)	31 (29.5%)
Female (%)	73 (69.5%)
Non-binary (%)	2 (1.0%)
**Age**	
Mean (SD)	23.65 (± 8.25)
**Region**	
North East	46 (43.8%)
Scotland	20 (19.0%)
Yorkshire	20 (19.0%)
West Midlands	13 (12.4%)
South East	6 (5.7%)
South West	1 (1.0%)
**Overall number of student paramedics that can speak one or more foreign languages at any level**	56 (53.3%)
**Most common languages spoken at any level**	
French	27 (48.2%)
Spanish	20 (35.7%)
German	12 (21.4%)
**Language ability of participants who can speak a foreign language**	(n = 56)
Beginner (CEFR A1–A2)	36 (64.3%)
Intermediate (CEFR B1–B2)	16 (28.6%)
Advanced (CEFR C1–C2)	4 (7.1%)
**Number of student paramedics who had studied a foreign language as part of their university learning**	(n = 102)
Yes	0 (0%)
No	102 (100%)
**Response to ‘Would you want to study an optional 10-credit foreign-language module alongside your paramedic course?’**	(n = 104)
Yes	60 (57.7%)
No	18 (17.3%)
Maybe	26 (25.0%)

The languages section of the survey captured that over half of the sample could speak a foreign language to at least a basic level or above and that the most commonly known foreign languages were those originating in Europe. There were a limited number of student paramedics who could speak a foreign language at an intermediate or advanced level. The most common languages spoken were French (48.2%), Spanish (35.7%) and German (21.4%). All participants (100%) reported that they had not had the opportunity to study a foreign language during their time at university. Last, 57.7% of the sample agreed that if they had the opportunity to study an additional 10-credit foreign-language module at university, they would do so.

## Discussion

This exploratory study collected data to understand the number of student paramedics who could speak a foreign language and which foreign languages were known and spoken among this population. As there is no current data exploring the foreign-language skills of student paramedics in the UK, this study has provided low-quality data to pioneer this area of exploration.

### Interpretation

The data showed that over half of respondents could speak a foreign language to some level, with the majority self-reporting skills between A1 and A2 (beginner level). This is a positive finding, as previous research indicates that foreign-language skills can improve communication, enhance cognitive abilities and lead to improved academic performance (Bak et al., 2016; Klimova, 2018; Woll & Wei, 2019). As this is a new area of study, with no current published data to explore student paramedic populations, we cannot determine whether these results are similar to other paramedic education programmes, both nationally and internationally.

To expand the discussion further, the literature highlights the need for healthcare education programmes to incorporate cultural competence simulation training and to broaden the scope of medical training to address culturally challenging encounters (Goth & Jensen, 2015; Skjerve et al., 2023). In this study, the majority of respondents expressed a desire to study a foreign language; however, current UK paramedic programmes lack the structure to facilitate language learning as part of the curriculum. There are examples of UK universities implementing extra-curricular language courses that paramedic faculties could investigate (Newcastle University 2023; University of Bristol, 2024) to inform language-learning programmes for student paramedics. Another potential solution could be to encourage self-directed learning, as previous research shows that registered and student paramedics can be committed to this approach (Alwidyan et al., 2022; Bryant et al., 2023; Williams et al., 2012). Furthermore, integrating foreign-language interactions into simulation training could be beneficial to applying this learning and to students’ overall communication skills (Blackmore et al., 2017; Mangan et al., 2022; Skjerve et al., 2023).

Last, it is important to mention that the current evidence shows that ethnic minorities are not being adequality represented in the current paramedic workforce, both nationally and internationally (Farquharson et al., 2017; Johnston et al., 2024). Focusing on improved recruitment and retention of ethnic minorities onto student paramedic programmes could increase the presence of foreign-language abilities within the ambulance workforce.

### Limitations

A key part of this exploratory study was the importance of capturing a wide sample that represented the student paramedic population effectively and authentically. However, despite the release of the study within 24 institutions, respondent uptake was low. On reflection, the survey may have encountered both self-selection and response bias, as individuals who speak foreign languages are more likely to be inclined to participate in a survey focused on foreign-language skills. This could have skewed the sample, as those without foreign-language skills might have been less motivated to respond, thus not providing a fully representative sample of the student paramedic population. However, regarding the aspect of collecting data that directly answered the aim and objectives of the research, the online survey approach with closed questions worked effectively.

To improve this research design, diversifying recruitment strategies beyond institutional channels to include targeted outreach via professional associations and social media could broaden participant demographics and reduce self-selection bias (Baker et al., 2022; Darko et al., 2022). Additionally, implementing recompensation for time given using gift cards or certificates could attract a more representative sample by encouraging participation from those less inclined to respond initially. Furthermore, a deeper analysis of the demographic may reveal trends in student paramedics that identify groups that may be more or less likely to have foreign-language skills.

### Future research

This exploratory study has shown that the research design could capture the foreign-language skill data of student paramedics, but a future project would need to improve recruitment via the methods mentioned previously to ensure reliability and validity in the data. The author plans to conduct a larger study, using a similar, but adapted approach, to quantify the foreign-language abilities of student paramedics. This could be used in conjunction with patient-focused research exploring their experience of language barriers when accessing pre-hospital care in the UK. Understanding both of these areas could inform an interventional programme to improve foreign-language skills of student paramedics, and of the wider ambulance workforce in the UK.

## Conclusion

This exploratory study captured foreign-language skills among student paramedics in the UK. The findings highlight a prevalent but limited level of foreign-language proficiency, particularly in European languages such as French, Spanish and German. However, the study faced significant challenges, including low respondent uptake and potential biases, such as self-selection, which limit the generalisability of the findings. Moving forward, addressing these methodological shortcomings through improved recruitment strategies is essential. This groundwork is pivotal for laying a foundation for future research that can provide robust insights into language capabilities among the paramedic workforce, facilitating targeted interventions to improve foreign-language competency in ambulance personnel.

## Acknowledgements

The author would like to thank student paramedic Ali Al-Omari for his contribution to the study. Ali completed administrative tasks, such as compiling the list of contacts for participant recruitment.

## Guarantor statement

OF designed and conceptualised the study, and is responsible for the methodology, data collection, data analysis and manuscript write-up. OF acts as the guarantor for this article.

## Conflict of interest

None declared.

## Ethics

Ethical approval was gained from the University of Sunderland (University of Sunderland ethics reference number: 024975). Written informed consent was gained in the survey.

## Funding

None.
